# Supplementary material for: TBK1 restricts IRGQ-mediated autophagy
Source: Nat Commun. 2026 May 13;17:4335. doi: 10.1038/s41467-026-73005-3 (PMC13172515; doi:10.1038/s41467-026-73005-3)
Supplement: Supplementary file 1 — Supplementary Information [file 41467_2026_73005_MOESM1_ESM.pdf]

Supplementary Information

TBK1 restricts IRGQ-mediated autophagy

Uxia Gestal-Mato[1], Pauline Lascaux[1], Sergio Alejandro Poveda-Cuevas[1,2], Alberto Cristiani[1,2], Belinda Camp[3], Mahyar Aghapour[3], Aparna Viswanathan Ammanath[1], Ramachandra M. Bhaskara[1,2,5], Ivan Dikic[1,2,4,5*], Lina Herhaus[1,3*]

[1] Institute of Biochemistry II, Goethe University Frankfurt, Medical Faculty, Theodor-Stern-Kai 7, 60590 Frankfurt am Main, Germany

[2] Buchmann Institute for Molecular Life Sciences, Goethe University Frankfurt, Riedberg Campus, Max-von-Laue-Straße 15, 60438 Frankfurt am Main, Germany

[3] Helmholtz Zentrum für Infektionsforschung GmbH, Inhoffenstraße 7, 38124 Braunschweig, Germany

[4] Max Planck Institute of Biophysics, Goethe University Frankfurt, Riedberg Campus, 60438 Frankfurt am Main, Germany

[5] IMPRS on Cellular Biophysics, Max-von-Laue Str. 3, 60438, Frankfurt am Main, Germany

* Correspondence should be addressed to: lina.herhaus@helmholtz-hzi.de, dikic@biochem2.uni-frankfurt.de

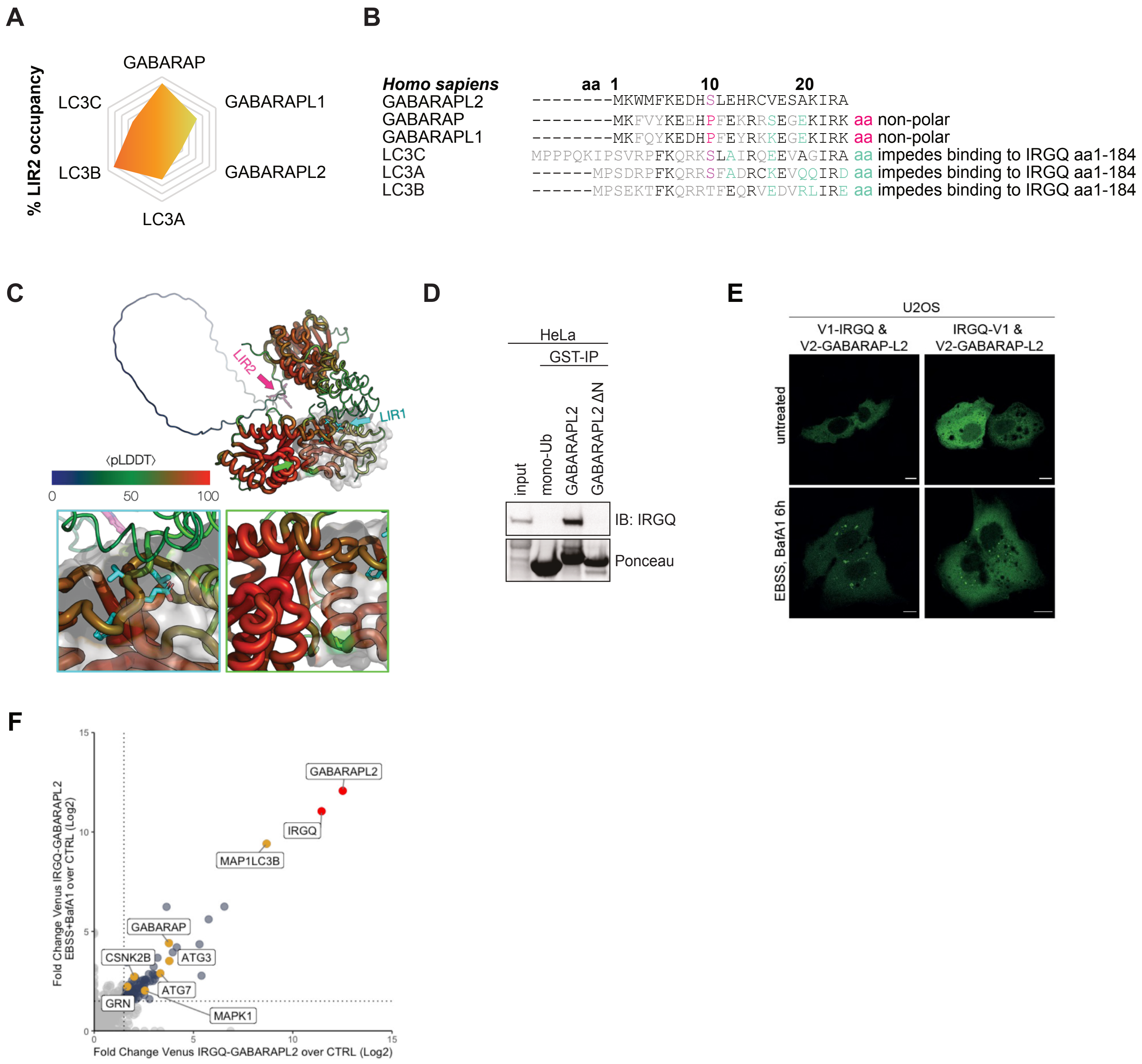

Figure S1: (A) Percentage of LIR2-LDS binding mode as the predicted complex between IRGQ-hATG8 in the 25 top-ranked AlphaFold2-multimer models from previous study16. **(B)** Sequence alignment of hATG8 N-terminals highlighting amino acids that would impede binding to IRGQ, in red polar residues, in green residues potentially causing steric clashes. **(C)** Top-ranked AlphaFold2 model for the IRGQ-GABARAPL2 complex. Confidence on local predicted structure (pLDDT) is averaged over top 100 models and mapped onto the structure (rainbow) GABARAPL2 is represented as a gray surface. Arrows indicate three key regions of IRGQ: LIR1 (cyan), LIR2 (pink), and switch II (green). Bottom panels show zoom-up of the LIR-LDS (left) and switch I/II protein-protein interfaces (right, ⟨pLDDT⟩ > 80). **(D)** SDS-PAGE and Western blot of an *in vitro* GST-pulldown using purified GST-mono-ubiquitin, GST-GABARAPL2 or GST-GABARAPL2 ΔN incubated with HeLa cell extract. **(E)** Immunofluorescence of U2OS cells, transiently expressing Vn-IRGQ and Vn-GABARAPL2, or IRGQ-Vn and GABARAPL2-Vc. Autophagy was induced by the addition of EBSS for 6 hours and lysosomal degradation was blocked by BafA1 (200 nM) treatment. **(F)** Scatter plot representing the Student's T-test difference from Vn-IRGQ-Vc-GABARAPL2 over VnVc-ORF IPs in Basal conditions and the Student's T-test difference from Vn-IRGQ-Vc-GABARAPL2 over VnVc-ORF IPs upon autophagy induction. The baits IRGQ and GABARAPL2 are marked in red, significant interaction partners in blue and autophagy-related proteins marked in yellow.

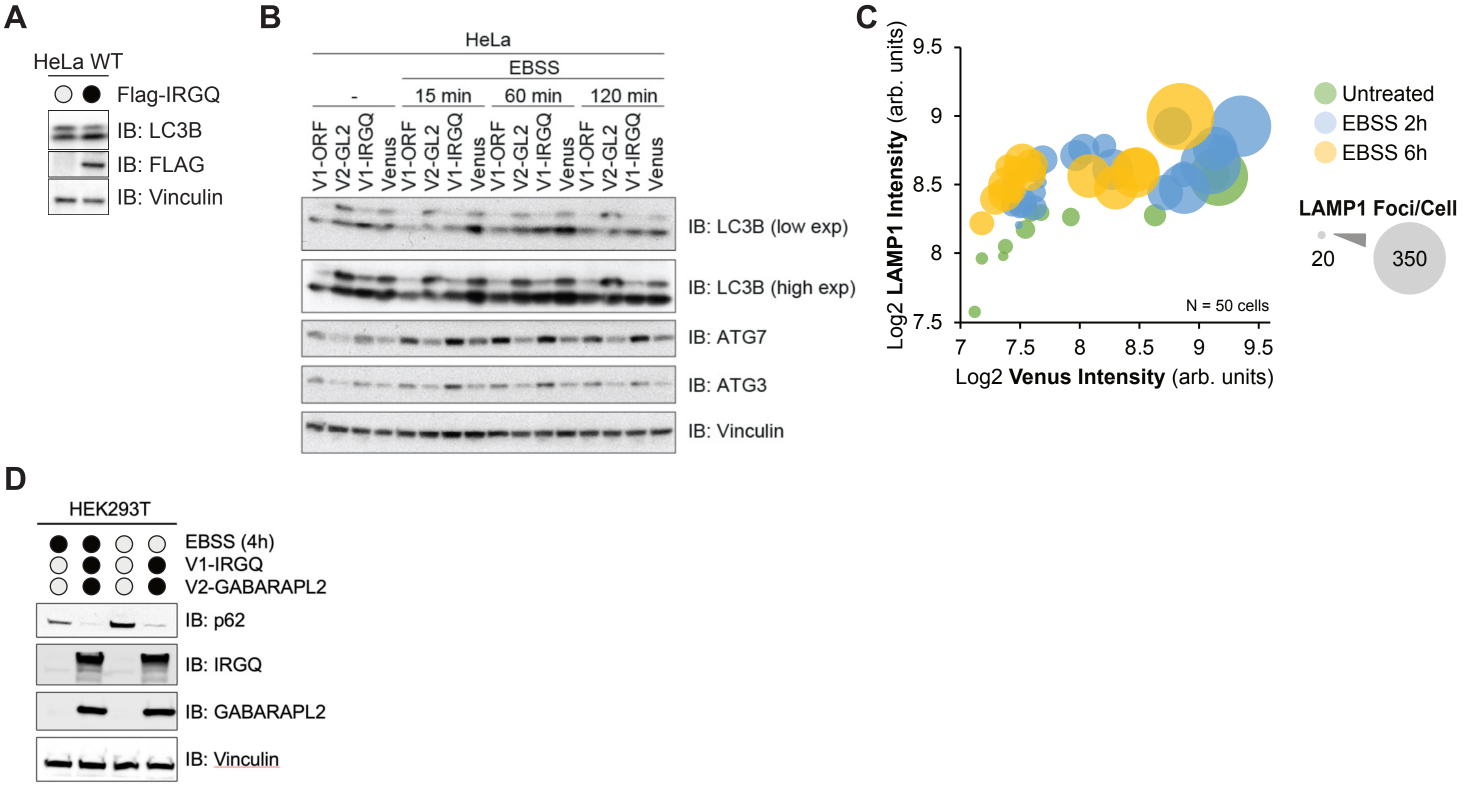

Figure S2: (A) SDS-PAGE and Western blot of HeLa cell lysates expressing transfected Flag-IRGQ. **(B)** SDS-PAGE and Western blot of HeLa cell lysates stably expressing V1-ORF, V2-GABARAPL2, V1-IRGQ or V1-IRGQ & V2-GABARAPL2 (Venus). Cells were either left untreated or treated with EBSS for indicated time points. **(C)** Scatter plot of ImageJ quantification from (D). Single cells were annotated as ROIs and intensities and puncta for LAMP1 and Venus were measured. RawIntDen values are plotted as Log2. Colors indicate the treatment and size of bubbles indicate the number of LAMP1 puncta per cell; n=50 cells. **(D)** SDS-PAGE and Western blot of HEK293T cell lysates with transfected V1-IRGQ and V2-GABARAPL2 untreated or treated with EBSS (4h).

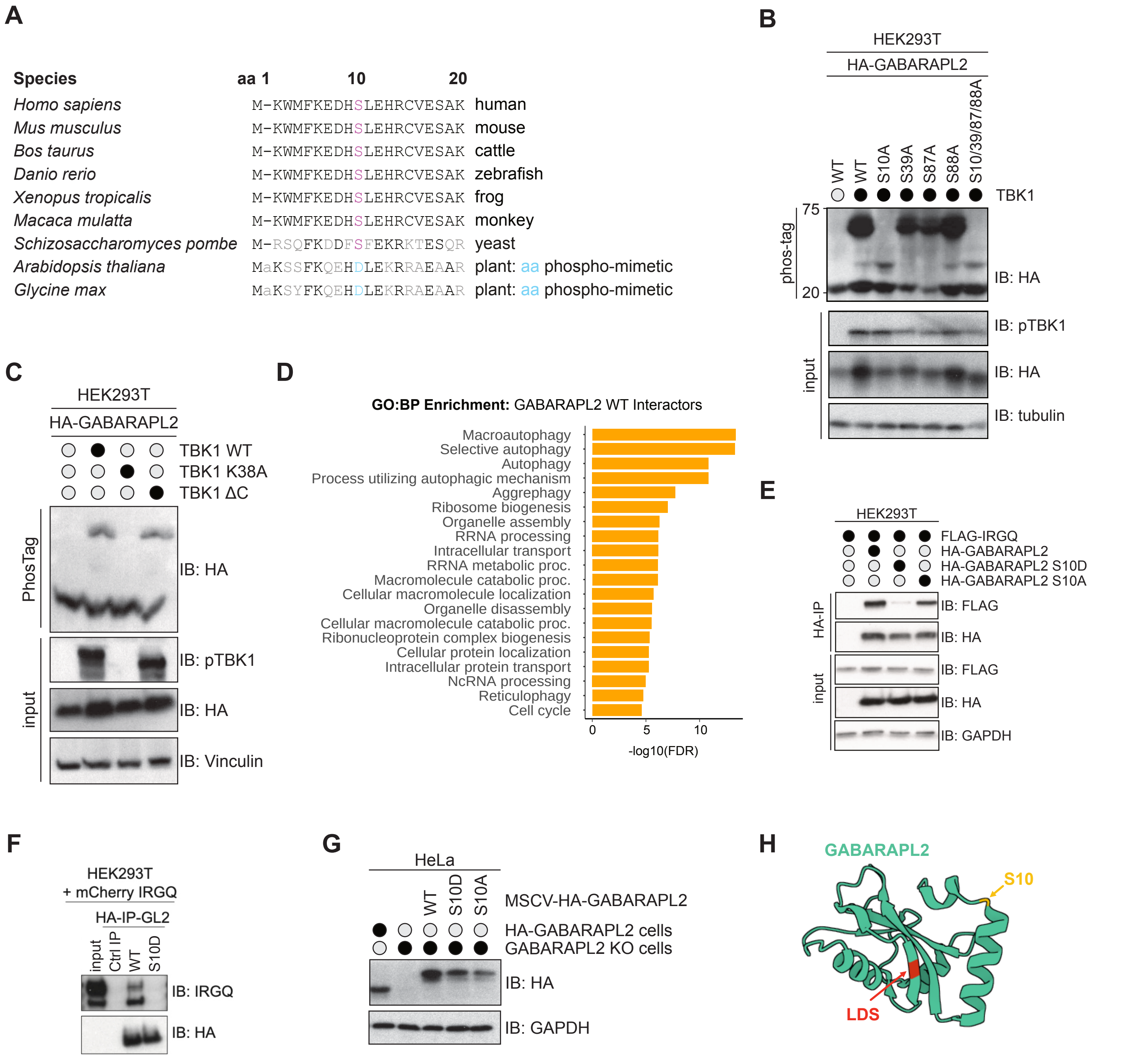

Figure S3: (A) Sequence alignment from multiple species for GABARAPL2 N-terminals (aa1-20) highlighted in red S10 conserved among the comparisons and in blue diverted amino acids in plants (D, phosphomimetic). **(B)** SDS-PAGE and Western blot of phos-tag gel with HEK293T cell lysates. Cells were transfected with TBK1 and HA-GABARAPL2 WT and mutants. **(C)** SDS-PAGE and Western blot of phos-tag gel with HEK293T cell lysates. Cells were transfected with HA-GABARA-L2 WT and TBK1 WT, TBK1 K38A (kinase inactive) and TBK1 ΔC-terminal coiled-coil region. **(D)** Gene Ontology enrichment of Biological Processes for HA-GABARAPL2 WT interactors, enrichment done with ShinyGO 0.77. **(E)** SDS-PAGE and Western blot of HEK293T cell lysates and HA-IPs. Cells were transfected with Flag-IRGQ, HA-GABARAPL2 WT, S10D or S10A and lysates used for HA-IPs. **(F)** SDS-PAGE and Western blot of HEK293T cell lysates and HA-IPs. Cells were transfected with mCherry-IRGQ and HA-GABARAPL2 WT or S10D and lysates used for HA-IPs. **(G)** SDS-PAGE and Western blot of HeLa GABARAP-L2 KO cells reconstituted with WT, S10A or S10D HA-GABARAPL2. **(H)** Crystal structure of GABARAPL2 (PDB:4CO7) with S10 marked in yellow and the LIR docking site (LDS) marked in red.

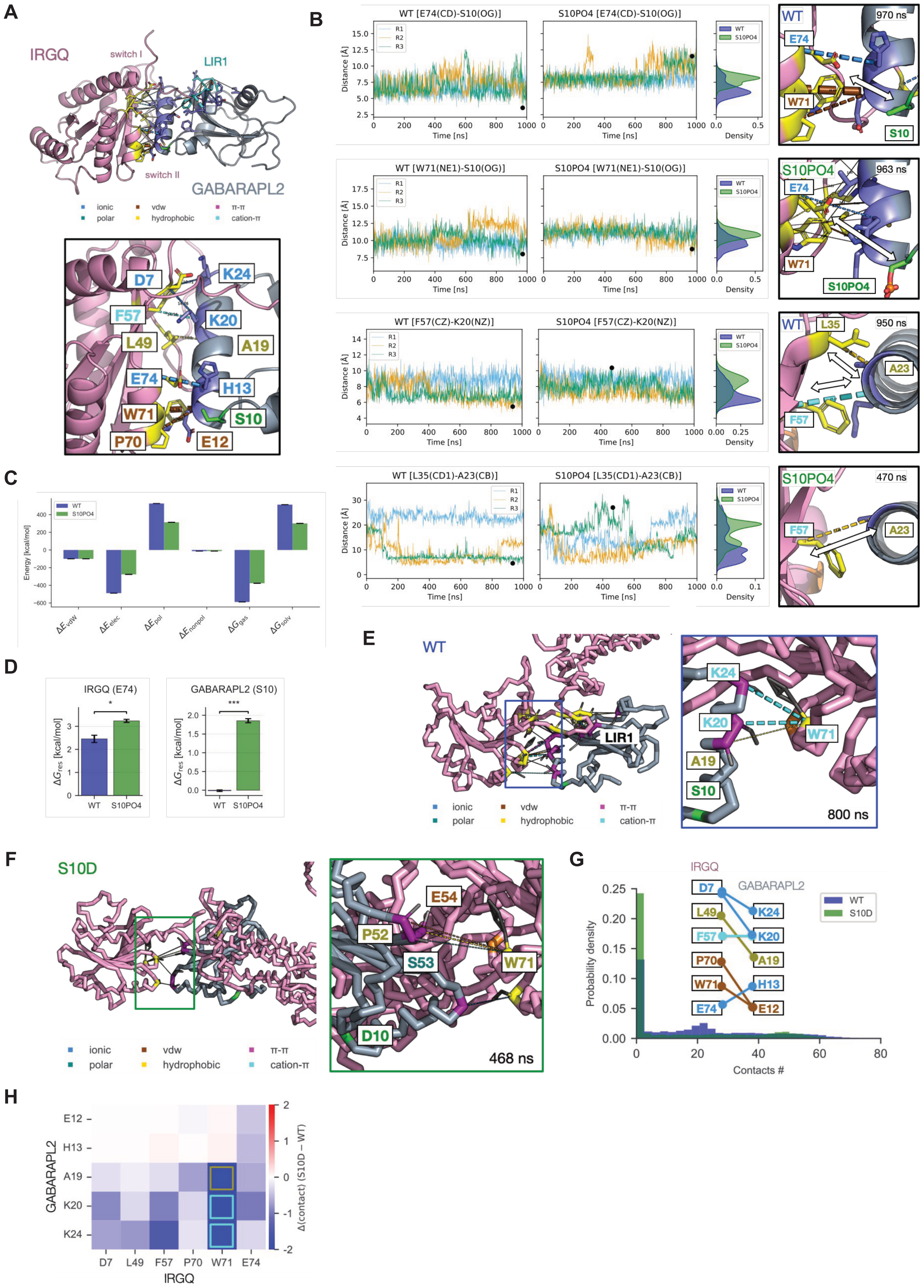
A
IRGQ
switch I
LIR1
switch II
GABARAPL2
ionic
polar
vdw
hydrophobic
π-π
cation-π
D7
K24
F57
K20
L49
A19
E74
H13
W71
S10
P70
E12
B
WT [E74(CD)-S10(OG)]
S10PO4 [E74(CD)-S10(OG)]
R1
R2
R3
WT
S10PO4
Distance [Å]
Time [ns]
Density
970 ns
WT [W71(NE1)-S10(OG)]
S10PO4 [W71(NE1)-S10(OG)]
963 ns
WT [F57(CZ)-K20(NZ)]
S10PO4 [F57(CZ)-K20(NZ)]
950 ns
L35
A23
WT [L35(CD1)-A23(CB)]
S10PO4 [L35(CD1)-A23(CB)]
470 ns
C
Energy [kcal/mol]
ΔE_{vdW}
ΔE_{elec}
ΔE_{pol}
ΔE_{nonpol}
ΔG_{gas}
ΔG_{solv}
D
IRGQ (E74)
GABARAPL2 (S10)
ΔG_{res} [kcal/mol]
E
WT
LIR1
800 ns
F
S10D
E54
P52
S53
D10
468 ns
G
IRGQ
GABARAPL2
WT
S10D
Probability density
Contacts #
H
GABARAPL2
IRGQ
Δ(contact) (S10D – WT)

Figure S4: (A) Xray model (8Q6Q) of the N-terminal domain (pink) of IRGQ containing LIR1 (186-189, cyan) in complex with GABARAPL2 (grey). Helix 2 of GBRL2 (purple) makes contacts within a pocket formed by IRGQ switch I (partially disordered), switch II, and the linker spanning LIR1 site and the N-terminal G-domain (bottom, zoom-up). Key ionic interactions across this interface (>10 residue pair-wise contacts) between IRGQ1-186 (yellow) and GBRL2 (purple) contribute to the complex stability. **(B)** Phosphorylation of GABARAPL2 destabilizes the IRGQ-GBRL2 switch I and II interface. Time series of distances between interacting residue pairs estimated from atomistic MD simulations (3 replicates x 1000 ns) of WT complex (left), and the phosphorylated complex (middle, S10-PO4). Comparison of density distributions of distances (WT vs. S10-PO4) across select interface residue-pairs (top to bottom) for E74-S10(OG), W71(NE1)-S10(OG), F57(CZ)-K20(NZ), and L35(CD1)-A23(CB). (right) Selected snapshots (indicated times) corresponding to the complex simulations showing the structural rearrangements at the interface for S10-PO4, indicating destabilization. Distances are shorter in the WT complex in comparison to S10-PO4. **(C)** MMPBSA energy computation and its decomposition into various terms shows relative contributions to the total binding energy of the IRGQ-GBRL2 complex (WT, blue; S10PO4, green). S10-PO4 complex displays an altered balance between electrostatic and solvation components, consistent with the structural reorganization of the interface induced by excess negative charge. **(D)** Residue-wise energy decomposition shows destabilizing effects. *** $p < 0.001$; * $p < 0.05$. **(E,F)** Representative snapshots from coarse-grained Go-Martini 3.0 simulations of IRGQ-GABARAPL2 complex (WT vs. S10D). Representative snapshot from WT complex simulations (15 replicates, 1000 ns each) showing preservation of the native interface. The LIR1 region remains stably engaged with GABARAPL2, and key interfacial residues (including K24, K20, A19, S10, and W71) maintain persistent contacts (right, zoom-up). By contrast, the S10D complex exhibits a disrupted native interface. The presumed interface residues mediate interactions with alternative sites on GABARAPL2, causing instability. **(G)** Distributions of the number of interface contacts in WT (blue) exhibit consistently higher interactions in comparison to S10D (green). **(H)** Heatmap showing the difference in pairwise residue–residue contact averages between WT and S10D (Δcnts. = $C_{ijS10D} - C_{ijWT}$). Most interface residue pairs strongly interact in the WT complex in comparison to S10D, consistent with altered electrostatic effects even in CGMD runs.

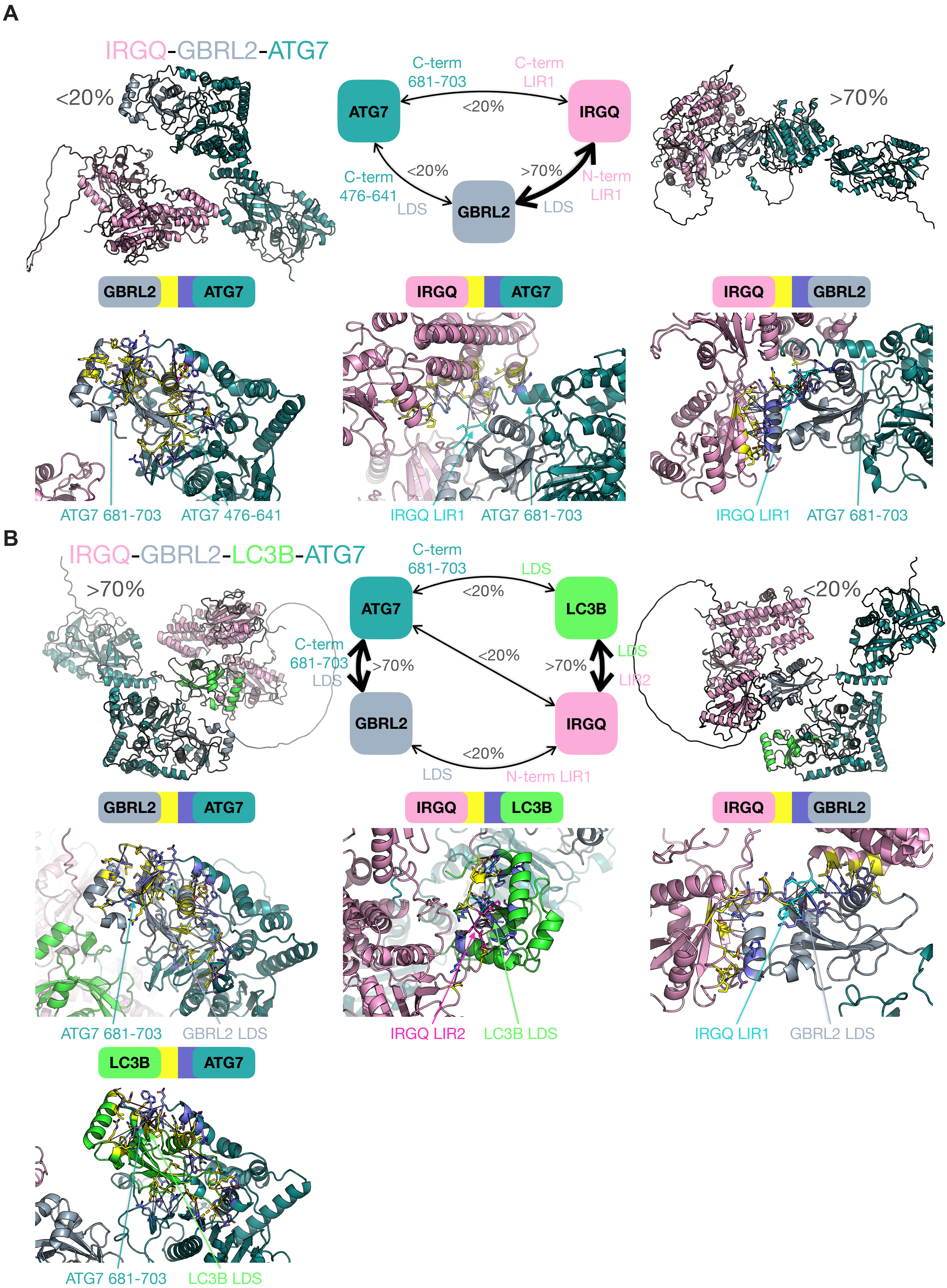

Figure S5: (A) AF2 multimers show differential IRGQ-hATG8 binding modes. Predicted IRGQ-GBRL2-ATG7 complexes display IRGQ interacting with GBRL2 in > 70% of the models (top right), while < 20% of the models represent GBRL2 interacting with ATG7 N- term (top left). Bottom panels display the pairwise interfaces (interfacing regions are coloured in yellow and violet for the first and second protein). **(B)** Predicted IRGQ-GBRL2-LC3B-ATG7 complexes display > 70% of models in which ATG7 interacts with GBRL2 and IRGQ interacts with LC3B (top left), while < 20% of the models present exchanged interactions, with ATG7 interacting with LC3B and IRGQ interacting with GBRL2 (top right). Bottom panels display the pairwise interfaces (interfacing regions are coloured inyellow and violet for the first and second protein).

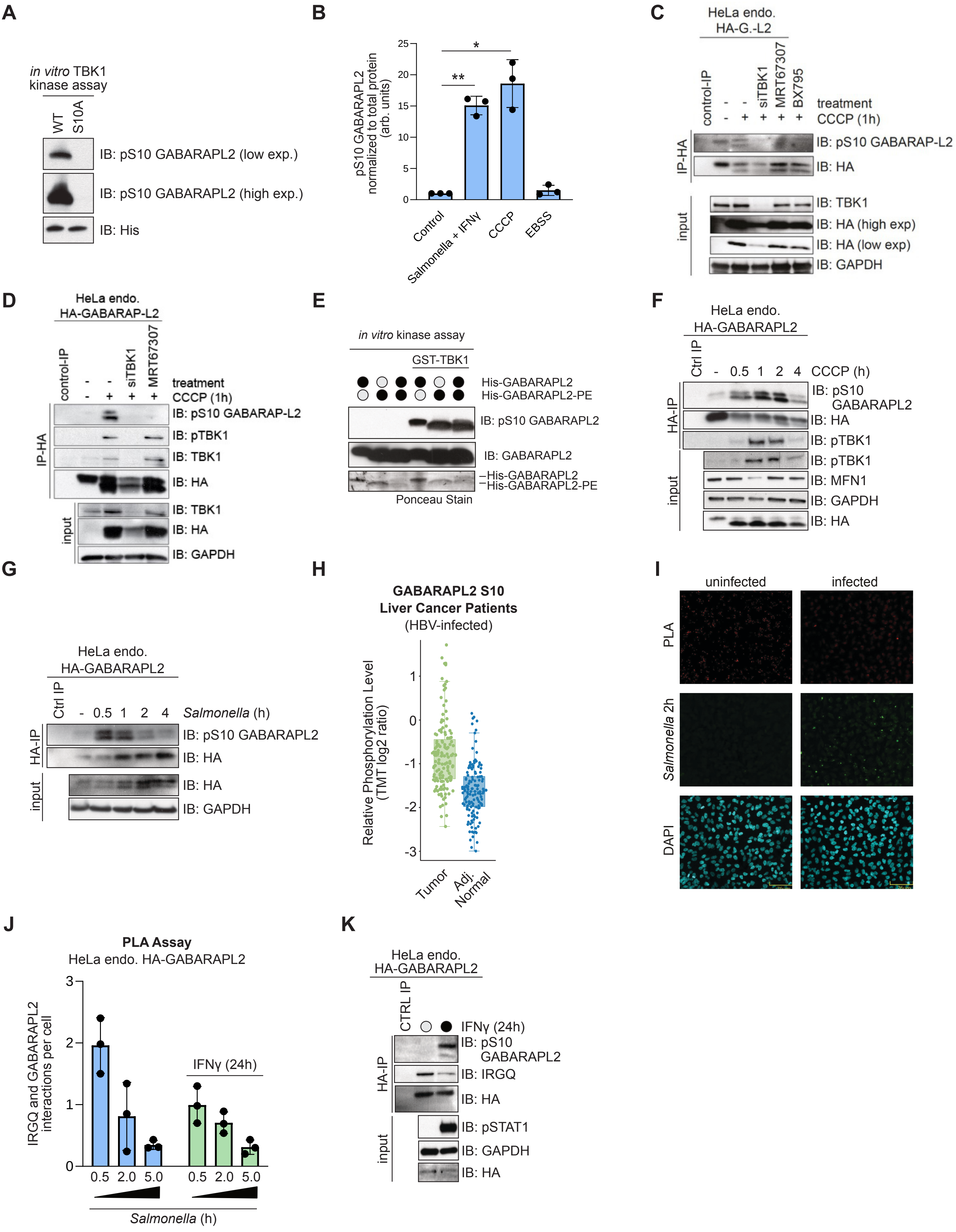
A
in vitro TBK1 kinase assay
WT
S10A
IB: pS10 GABARAPL2 (low exp.)
IB: pS10 GABARAPL2 (high exp.)
IB: His
B
pS10 GABARAPL2 normalized to total protein (arb. units)
25
20
15
10
5
0
*
**
Control
Salmonella + IFNγ
CCCP
EBSS
C
HeLa endo. HA-G.-L2
control-IP
siTBK1
MRT67307
BX795
treatment
CCCP (1h)
IP-HA
IB: pS10 GABARAP-L2
IB: HA
input
IB: TBK1
IB: HA (high exp)
IB: HA (low exp)
IB: GAPDH
D
HeLa endo. HA-GABARAP-L2
control-IP
siTBK1
MRT67307
treatment
CCCP (1h)
IP-HA
IB: pS10 GABARAP-L2
IB: pTBK1
IB: TBK1
IB: HA
input
IB: TBK1
IB: HA
IB: GAPDH
E
in vitro kinase assay
GST-TBK1
His-GABARAPL2
His-GABARAPL2-PE
IB: pS10 GABARAPL2
IB: GABARAPL2
His-GABARAPL2
His-GABARAPL2-PE
Ponceau Stain
F
HeLa endo. HA-GABARAPL2
Ctrl IP
- 0.5 1 2 4 CCCP (h)
HA-IP
IB: pS10 GABARAPL2
IB: HA
IB: pTBK1
input
IB: pTBK1
IB: MFN1
IB: GAPDH
IB: HA
G
HeLa endo. HA-GABARAPL2
Ctrl IP
- 0.5 1 2 4 Salmonella (h)
HA-IP
IB: pS10 GABARAPL2
IB: HA
input
IB: HA
IB: GAPDH
H
GABARAPL2 S10
Liver Cancer Patients
(HBV-infected)
Relative Phosphorylation Level (TMT log2 ratio)
1
0
-1
-2
-3
Tumor
Adj. Normal
I
uninfected
infected
PLA
Salmonella 2h
DAPI
J
PLA Assay
HeLa endo. HA-GABARAPL2
IRGQ and GABARAPL2 interactions per cell
3
2
1
0
IFNγ (24h)
0.5 2.0 5.0
0.5 2.0 5.0
Salmonella (h)
K
HeLa endo. HA-GABARAPL2
CTRL IP
IFNγ (24h)
HA-IP
IB: pS10 GABARAPL2
IB: IRGQ
IB: HA
input
IB: pSTAT1
IB: GAPDH
IB: HA

Figure S6: (A) SDS-PAGE and Western blot of *in vitro* TBK1 kinase assay with His-GABARAPL2 WT or S10A as substrate to test the pS10 GABARAPL2 antibody specificity. **(B)** ImageJ quantification from (Fig. 4A) of pS10 GABARAPL2, normalized to total GABARAPL2 protein. Data are presented as the mean with error bars indicating the s.d. Statistical significance of differences between experimental groups was assessed with Student's T-test. Differences with $p<0.05$ are annotated as * and $p<0.01$ are annotated as **; n=3. **(C)** SDS-PAGE and Western blot of HA-IPs from endogenously tagged HA-GABARAPL2 WT HeLa cells after treatment with TBK1 inhibitors (MRT67307 or BX795) or TBK1 siRNA knock-down, plus CCCP treatment (1h). **(D)** SDS-PAGE and Western blot of HA-IPs from endogenously tagged HA-GABARAPL2 WT HeLa cells after treatment with TBK1 inhibitor (MRT67307) or TBK1 siRNA knock-down, plus CCCP treatment (1h). **(E)** SDS-PAGE and Western blot of *in vitro* TBK1 kinase assay with His-GABARAPL2 and His-GABARAPL2-PE as substrate. **(F)** SDS-PAGE and Western blot of endogenously tagged HA-GABARAPL2 cells treated with 40 uM CCCP at specified timepoints. Lysates were used for HA-IP. **(G)** SDS-PAGE and Western blot of endogenously tagged HA-GABARAPL2 cells Infected with Salmonella (MOI:150) at specified timepoints. Lysates were used for HA-IP. **(H)** Relative Phosphorylation Level of GABARAPL2 S10 in HBV-derived liver samples from human samples. Data retrieved from www.cprosite.ccr.cancer.gov,[45]. **(I)** Images of PLA signal (red) from endogenous GABARAP-L2 and endogenous IRGQ. HeLa endogenous HA-GABARAPL2 cells were infected with *Salmonella* (GFP) for 2 hours and fixed cells were probed with the Duolink in situ PLA assay. HA and IRGQ only antibodies were used as negative controls to determine the background. **(J)** Yokogawa CQ1 quantification of average Duolink PLA signal from endogenous GABARAP-L2 and endogenous IRGQ. HeLa endogenous HA-GABARAPL2 cells were treated with 10 ng/ml IFNγ (24 hours), infected with Salmonella for indicated time points and fixed cells were probed with the Duolink in situ PLA assay. Data are presented as the mean with error bars indicating the s.d.; n=3; >3000 cells/condition. **(K)** SDS-PAGE and Western blot of endogenously tagged HA-GABARAPL2 cells treated with 10 ng/ml IFNγ (24 hours). Lysates were used for HA-IP.

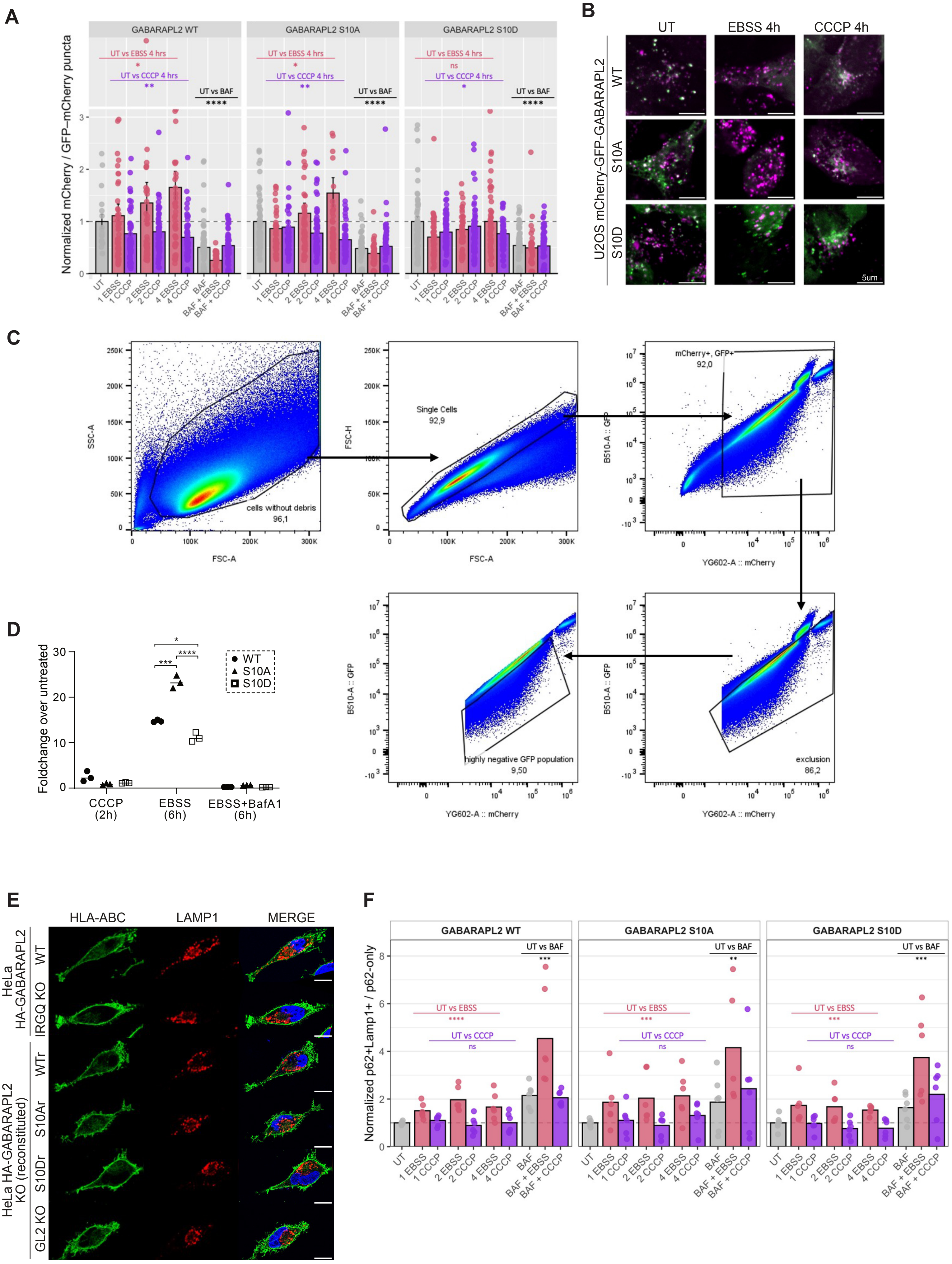
A
GABARAPL2 WT
GABARAPL2 S10A
GABARAPL2 S10D
UT vs EBSS 4 hrs
UT vs CCCP 4 hrs
UT vs BAF
ns
Normalized mCherry / GFP–mCherry puncta
UT
1 EBSS
1 CCCP
2 EBSS
2 CCCP
4 EBSS
4 CCCP
BAF
BAF + EBSS
BAF + CCCP
B
UT
EBSS 4h
CCCP 4h
U2OS mCherry-GFP-GABARAPL2
WT
S10A
S10D
5um
C
cells without debris
96,1
Single Cells
92,9
mCherry+, GFP+
92,0
highly negative GFP population
9,50
exclusion
86,2
FSC-A
SSC-A
FSC-H
B510-A :: GFP
YG602-A :: mCherry
D
Foldchange over untreated
WT
S10A
S10D
CCCP (2h)
EBSS (6h)
EBSS+BafA1 (6h)
E
HLA-ABC
LAMP1
MERGE
HeLa HA-GABARAPL2
WT
IRGQ KO
HeLa HA-GABARAPL2 KO (reconstituted)
WTr
S10Ar
S10Dr
GL2 KO
F
GABARAPL2 WT
GABARAPL2 S10A
GABARAPL2 S10D
UT vs BAF
UT vs EBSS
UT vs CCCP
ns
Normalized p62+Lamp1+ / p62-only
UT
1 EBSS
1 CCCP
2 EBSS
2 CCCP
4 EBSS
4 CCCP
BAF
BAF + EBSS
BAF + CCCP

Figure S7: (A) GABARAPL2-to-lysosome flux under stress is lost for the S10D phosphorylation mutant. Quantification of GABARAPL2-to-lysosome flux in cells expressing GABARAPL2 WT, S10A, or S10D. Each point represents the mean mCherry/GFP-mCherry puncta ratio per well and per biological replicate (four independent experiments, two wells per condition). Data were first normalized to the mean untreated (UT) signal averaged across WT, S10A, and S10D within each replicate, and then rescaled so that UT = 1 for all genotypes in the final plot. Conditions correspond to nutrient starvation (EBSS; 1-4 h), mitochondrial depolarization (40 µM CCCP; 1-4 h), or lysosomal inhibition (200 nM BAF for 4 h; ± EBSS or CCCP). One-sample Wilcoxon signed-rank with Holm p-value correction. **** $p < 0.0001$; *** $p < 0.001$; ** $p < 0.01$; * $p< 0.05$; n.s., not significant. **(B)** Immunofluorescence of cells expressing mCherry/GFP-GABARAPL2 WT, S10A, or S10D. Conditions correspond to nutrient starvation (EBSS; 1-4 h), mitochondrial depolarization (40 µM CCCP; 1-4 h), or lysosomal inhibition (200nM BAF for 4 h; ± EBSS or CCCP). **(C)** FACS analysis of of GABARAPL2-to-lysosome flux in cells expressing mCherry-GFP GABARAPL2 WT, S10A, or S10D. Each point represents the mean mCherry/GFP–mCherry puncta ratio per well and per biological replicate. Data is presented as fold change and normalized to the mean untreated cell line (WT, S10A or S10D, respectively). Conditions correspond to nutrient starvation (EBSS 6 h), mitochondrial depolarization (40 µM CCCP 2 h), or lysosomal inhibition (200nM BAF for 6 h + EBSS 6 h). n=3. **(D)** Immunofluorescence of HeLa GABARAPL2 WT, IRGQ KO or GABARAPL2 KO cells reconstituted with WT, S10A or S10D HA-GABARAPL2. Autophagy was induced by the addition of EBSS for 4 hours and Bafilomycin A1 (200 nM). Fixed cells were probed with endogenous LAMP1 and HLA-ABC antibodies. Scale bar: 10 µm. **(E)** p62-to-lysosome flux under stress conditions is independent of GABARAPL2 S10 phosphorylation. Quantification of p62-to-lysosome flux in GABARAPL2 WT, S10A, and S10D cells. Each point represents the normalized mean ratio per well and per biological repeat (three independent experiments, two wells per condition). Values were normalized to the untreated (UT) mean within each genotype and repeat. Conditions represent starvation (EBSS, 1-4 h), mitochondrial depolarization (40µM CCCP, 1-4 h), or lysosomal inhibition (200 nM BAF for 4 h; ± EBSS or CCCP). Grouped one-sample t-tests compared combined EBSS (E1-E4), CCCP (C1-C4), and BAF (BAF, EBSS + BAF, CCCP + BAF) groups to UT (= 1). **** $p < 0.0001$; *** $p < 0.001$;
** $p < 0.01$; * $p < 0.05$; n.s., not significant.
