# Supplementary material for: TBK1 restricts IRGQ-mediated autophagy
Source: Nat Commun. 2026 May 13;17:4335. doi: 10.1038/s41467-026-73005-3 (PMC13172515; doi:10.1038/s41467-026-73005-3)
Supplement: Supplementary file 2 — Reporting Summary [file 41467_2026_73005_MOESM2_ESM.pdf]

nature portfolio

Corresponding author(s): Lina Herhaus
Last updated by author(s): Mar 13, 2026

Reporting Summary

Nature Portfolio wishes to improve the reproducibility of the work that we publish. This form provides structure for consistency and transparency in reporting. For further information on Nature Portfolio policies, see our Editorial Policies and the Editorial Policy Checklist.

Statistics

For all statistical analyses, confirm that the following items are present in the figure legend, table legend, main text, or Methods section.

n/a	Confirmed	
☐	☒	The exact sample size (*n*) for each experimental group/condition, given as a discrete number and unit of measurement
☐	☒	A statement on whether measurements were taken from distinct samples or whether the same sample was measured repeatedly
☐	☒	The statistical test(s) used AND whether they are one- or two-sided<br>*Only common tests should be described solely by name; describe more complex techniques in the Methods section.*
☐	☒	A description of all covariates tested
☐	☒	A description of any assumptions or corrections, such as tests of normality and adjustment for multiple comparisons
☐	☒	A full description of the statistical parameters including central tendency (e.g. means) or other basic estimates (e.g. regression coefficient) AND variation (e.g. standard deviation) or associated estimates of uncertainty (e.g. confidence intervals)
☐	☒	For null hypothesis testing, the test statistic (e.g. *F*, *t*, *r*) with confidence intervals, effect sizes, degrees of freedom and *P* value noted<br>*Give P values as exact values whenever suitable.*
☒	☐	For Bayesian analysis, information on the choice of priors and Markov chain Monte Carlo settings
☒	☐	For hierarchical and complex designs, identification of the appropriate level for tests and full reporting of outcomes
☒	☐	Estimates of effect sizes (e.g. Cohen's *d*, Pearson's *r*), indicating how they were calculated

Our web collection on statistics for biologists contains articles on many of the points above.

Software and code

Policy information about availability of computer code

Data collection	Excel, CellProfiler 4.2.8, alpha fold
Data analysis	Excel, CellProfiler 4.2.8 and Prism softwares, R

For manuscripts utilizing custom algorithms or software that are central to the research but not yet described in published literature, software must be made available to editors and reviewers. We strongly encourage code deposition in a community repository (e.g. GitHub). See the Nature Portfolio guidelines for submitting code & software for further information.

Data

Policy information about availability of data

All manuscripts must include a data availability statement. This statement should provide the following information, where applicable:

- Accession codes, unique identifiers, or web links for publicly available datasets
- A description of any restrictions on data availability
- For clinical datasets or third party data, please ensure that the statement adheres to our policy

The data generated in this study are provided in the Supplementary Information/Source Data file. Source data are provided with this paper. The mass spectrometry proteomic data have been deposited to the ProteomeXchange Consortium via the PRIDE partner repository with the following dataset identifiers: IRGQ-GABARAPL2 complex interactome data - PXD066671, GABARAPL2 interactome - PXD066665.

Research involving human participants, their data, or biological material

Policy information about studies with human participants or human data. See also policy information about sex, gender (identity/presentation), and sexual orientation and race, ethnicity and racism.

Reporting on sex and gender	No human participants are involved in this study, hence this information has not been collected.
Reporting on race, ethnicity, or other socially relevant groupings	No human participants are involved in this study, hence this information has not been collected.
Population characteristics	No human participants are involved in this study, hence this information has not been collected.
Recruitment	No human participants are involved in this study, hence this information has not been collected.
Ethics oversight	No human participants are involved in this study, hence this information has not been collected.

Note that full information on the approval of the study protocol must also be provided in the manuscript.

Field-specific reporting

Please select the one below that is the best fit for your research. If you are not sure, read the appropriate sections before making your selection.

[X] Life sciences [ ] Behavioural & social sciences [ ] Ecological, evolutionary & environmental sciences

For a reference copy of the document with all sections, see nature.com/documents/nr-reporting-summary-flat.pdf

Life sciences study design

All studies must disclose on these points even when the disclosure is negative.

Sample size	Sample sizes were determined based on established standards in the field and our prior experience with the variability of these assays. Unless otherwise stated, each experiment was performed in technical triplicates and repeated as independent biological triplicates. This design reliably detects reproducible effect sizes observed in our preliminary data and previously published work. No formal statistical power calculation was performed; instead, sample numbers were chosen to ensure robustness while minimizing unnecessary experimental burden.
Data exclusions	Data were only excluded if predefined technical criteria were met (e.g., assay failure, contamination, or clear equipment malfunction). Exclusion criteria were established prior to data analysis.<br>No single data points were excluded from the analyses of a whole experiment.
Replication	All key findings were independently replicated. Each experiment was conducted in at least three biological replicates, with each biological replicate measured in technical triplicates. Replicated experiments produced consistent results unless otherwise indicated.
Randomization	Where applicable, samples and experimental conditions were allocated using randomization procedures to minimize bias (e.g., random assignment of samples to plate positions). For experiments where randomization was not relevant (e.g., in-vitro assays), samples were processed in parallel under identical conditions.
Blinding	Data collection and primary analysis were conducted with investigators blinded to group identity where feasible (e.g. Immunofluorescence analysis). In other settings blinding was not possible due to experimental constraints (e.g. Western Blots).

Reporting for specific materials, systems and methods

We require information from authors about some types of materials, experimental systems and methods used in many studies. Here, indicate whether each material, system or method listed is relevant to your study. If you are not sure if a list item applies to your research, read the appropriate section before selecting a response.

Materials & experimental systems

n/a	Involved in the study	
[ ]	[X]	Antibodies
[ ]	[X]	Eukaryotic cell lines
[X]	[ ]	Palaeontology and archaeology
[X]	[ ]	Animals and other organisms
[X]	[ ]	Clinical data
[X]	[ ]	Dual use research of concern
[X]	[ ]	Plants

Methods

n/a	Involved in the study	
[X]	[ ]	ChIP-seq
[ ]	[X]	Flow cytometry
[X]	[ ]	MRI-based neuroimaging

Antibodies

Antibodies used	anti-HA-tag (11867423001; Roche), anti-FlagM2-tag (F3165; Sigma), anti-GFP-tag (Living Colors 632592; Clontech), anti-His-tag (11922416001; Roche), anti-vinculin (V4505; Sigma), anti-TBK1 (#3013; Cell Signaling Technology), anti-pTBK1 (pS172; #5483; Cell Signaling Technology), anti-IRGQ (HPA043254; Sigma), anti-GAPDH (#2118; Cell Signaling Technology), anti-GABARAP-L2 (PM038; MBL), anti-LAMP1 (H4A3; DSHB), anti-p62 (M162-3; MBL), anti-IkBa (#9247 ; Cell Signaling Technology), anti-Histone H3 (ab1791, Abcam), anti-pSTAT1 (pY701; #7649; Cell Signaling Technology), anti-ATG3 (#3415 ; Cell Signaling Technology), anti-ATG7 (8558S, CST),anti-LC3B (PMO36; MBL), anti-ULK1 (8054S, CST), anti-MFN1 (14739S, CST), anti-pS10 GABARAP-L2 (was generated by immunGlobe®, a chemically synthesized peptide (GABARAP-L2 aa4-15) bearing a phosphate group at S10 (Ac-MFKEDH(pS)LEHRC-NH2) was used for immunization).
Validation	P-S10 GABARAPL2 antibody was validated by WB see Figure S6A. All other commercial antibodies were used in accordance with The manufacturer instructions.

Eukaryotic cell lines

Policy information about cell lines and Sex and Gender in Research

Cell line source(s)	HEK293T, Hela and U2OS cells were obtained from ATCC.
Authentication	None of the cell lines used where authenticated.
Mycoplasma contamination	All cell lines area negative for mycoplasma. Mycoplasma testing is done routinely.
Commonly misidentified lines (See ICLAC register)	N/A

Plants

Seed stocks	No plants are involved in this study, hence this information has not been collected.
Novel plant genotypes	No plants are involved in this study, hence this information has not been collected.
Authentication	No plants are involved in this study, hence this information has not been collected.

Flow Cytometry

Plots

Confirm that:

☒ The axis labels state the marker and fluorochrome used (e.g. CD4-FITC).

☒ The axis scales are clearly visible. Include numbers along axes only for bottom left plot of group (a 'group' is an analysis of identical markers).

☒ All plots are contour plots with outliers or pseudocolor plots.

☒ A numerical value for number of cells or percentage (with statistics) is provided.

Methodology

Sample preparation	T-REx mCherry-GFP GABARAP-L2 WT, S10A and S10D mutant U2OS were cultivated in cell culture flasks until ~80 % confluency and transferred to 6-well plates (500.000 cells/well) for the experiments (3 replicates/condition for every cell line). Cells were treated over night with doxycycline (final concentration: 1 µg/ml) to induce mCherry-GFP expression. Negative control was not induced. After induction, cells were washed once with PBS and treated as follows: untreated (stopped after 2 h), CCCP (40 µM, 2 h), EBSS (6 h) and EBSS + Bafilomycin (200 nM, 6 h). After treatments, cells were detached using trypsin/EDTA, centrifuged (5 min, 500 x g) and resuspended in 200 µl of FACS buffer (PBS + EDTA + FBS).
Instrument	FACS was performed directly at BD Symphony A5
Software	FlowJo software (version 10), Analysis was performed using GraphPad Prism software.
Cell population abundance	The mean fluorescence intensity (MFI) for mCherry and GFP was calculated for each sample. Fold changes were calculated and normalized to the mean of each untreated cell line (WT, S10A and S10D, respectively).

Gating strategy

Following singlet gating, cells were gated for high mCherry+ GFP+ cells using FlowJo software. Non-induced (no Doxycycline treatment) cells were used as a negative control.

☒ Tick this box to confirm that a figure exemplifying the gating strategy is provided in the Supplementary Information.
