# Supplementary material for: TBK1 restricts IRGQ-mediated autophagy
Source: Nat Commun. 2026 May 13;17:4335. doi: 10.1038/s41467-026-73005-3 (PMC13172515; doi:10.1038/s41467-026-73005-3)

Raw data (1): uncropped blots / membranes

1F

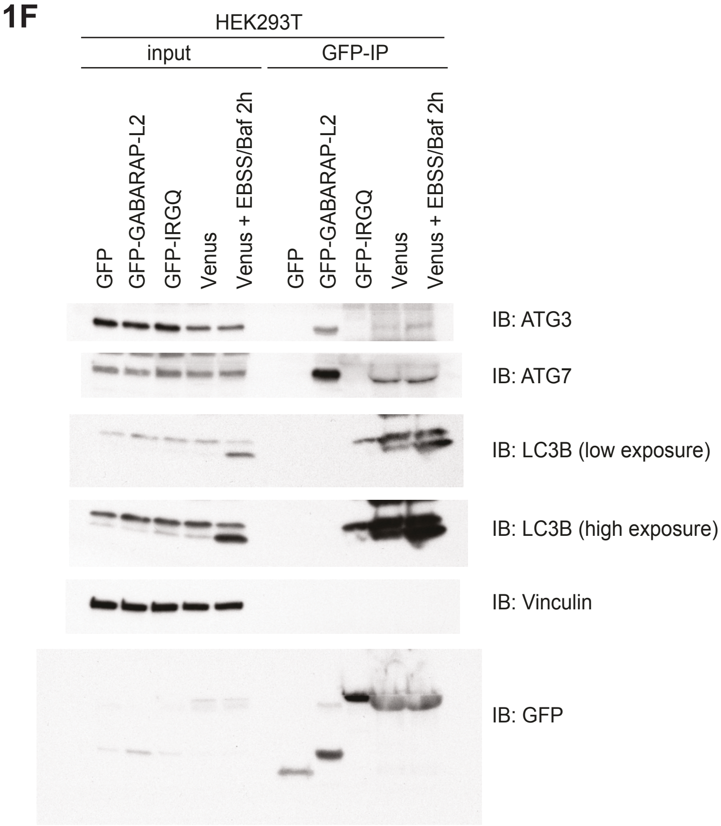

1G

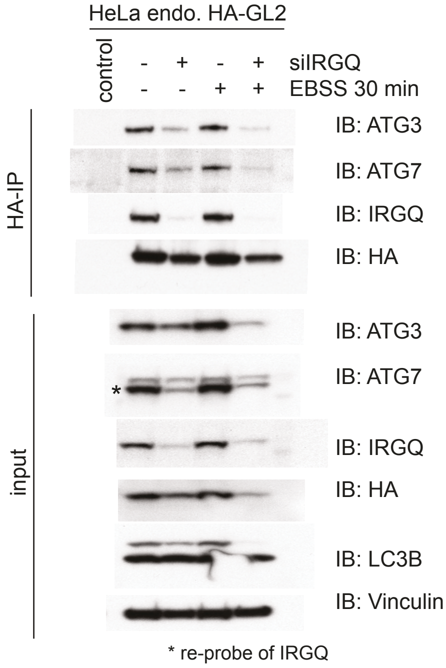

S1D

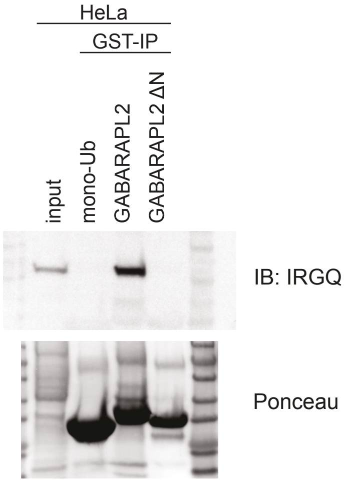

2A

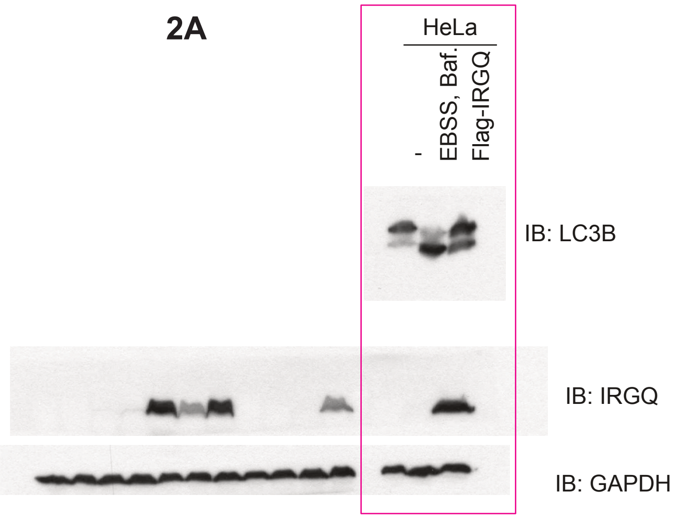

2B

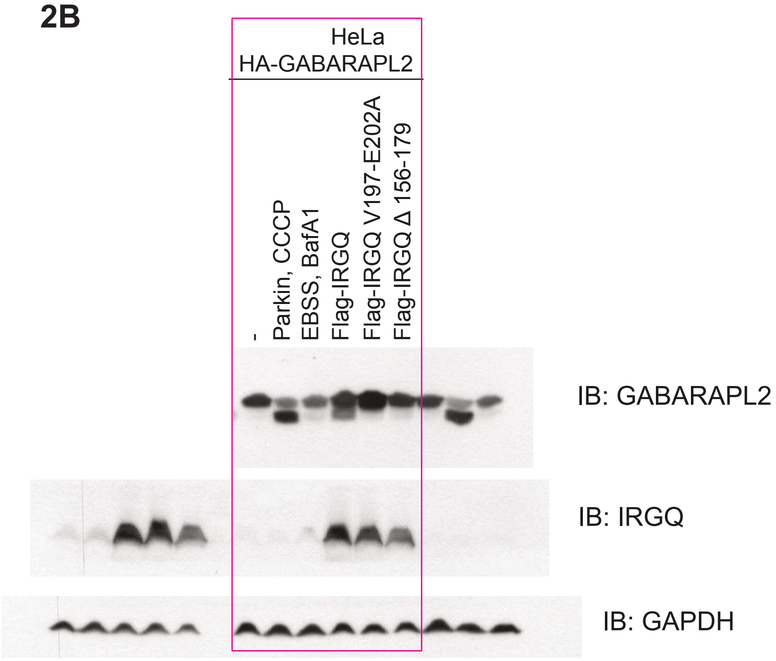

2C

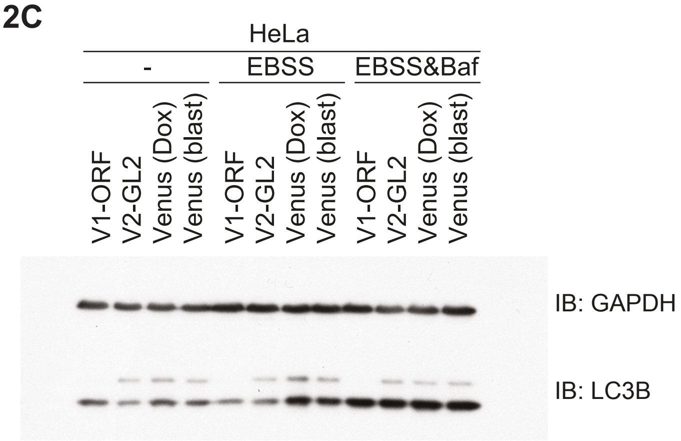

Raw data (2): uncropped blots / membranes

S2A

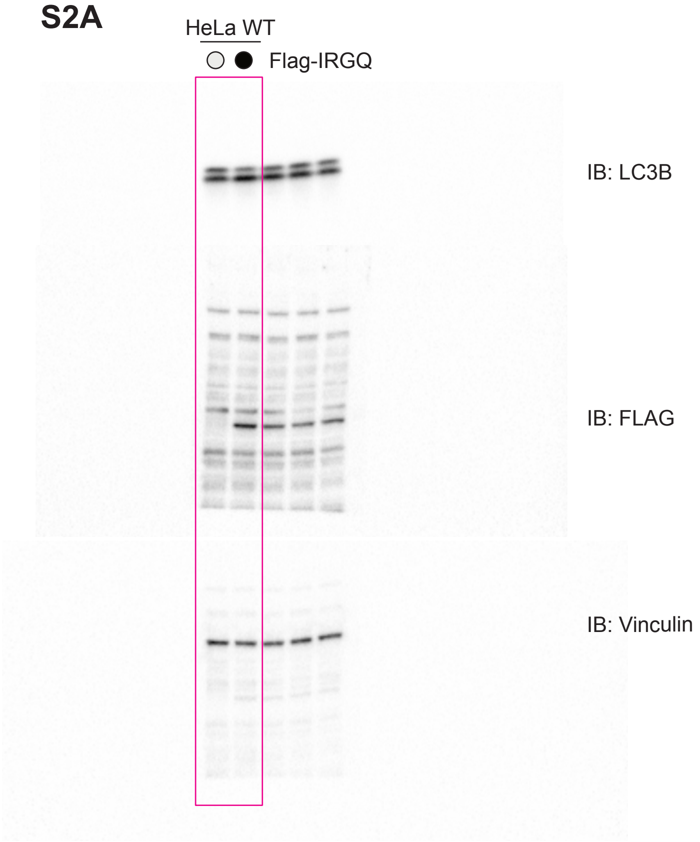

S2B

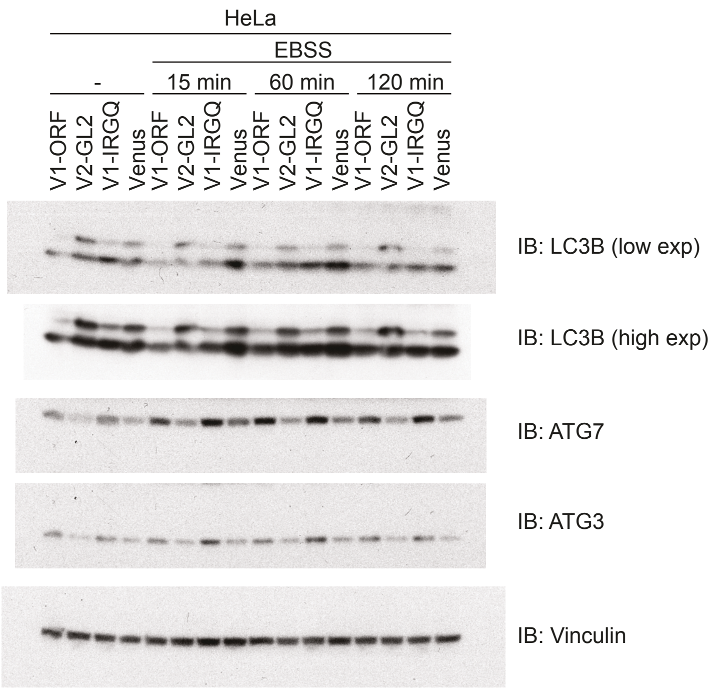

3B

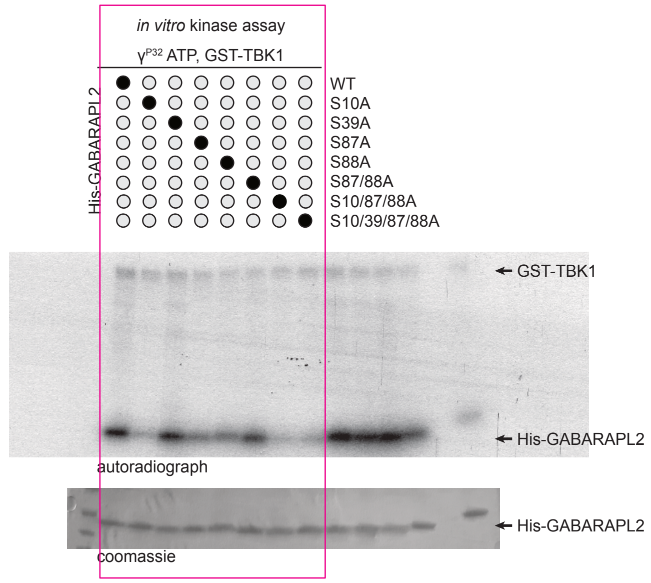

S2D

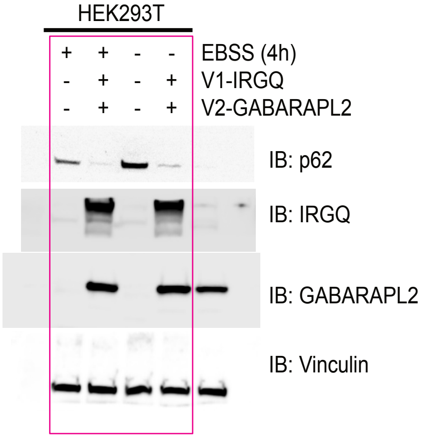

3F

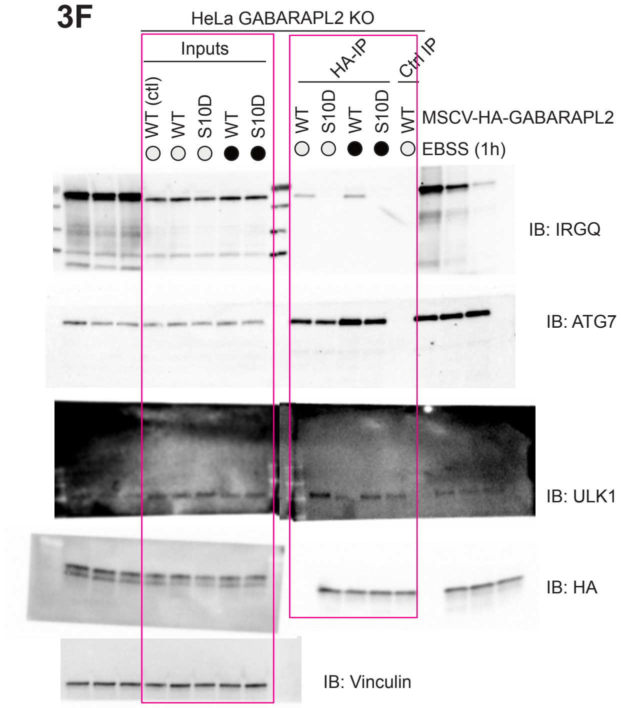

S3B

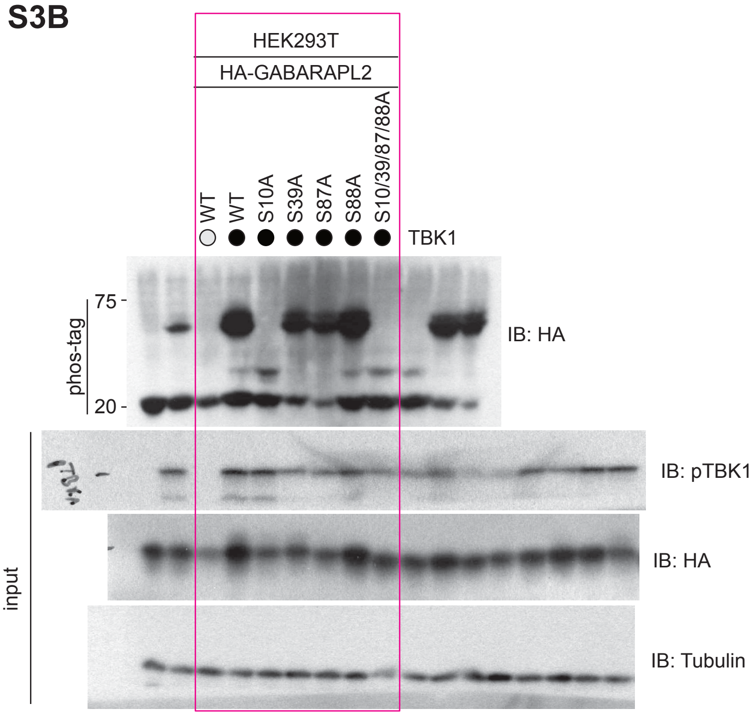

Raw data (3): uncropped blots / membranes

S3C

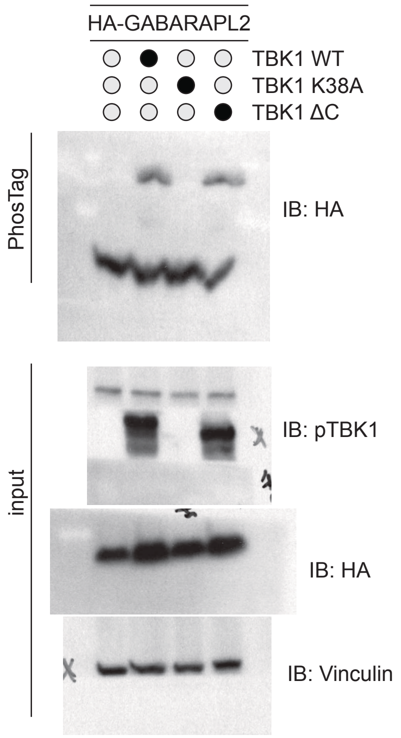

S3E

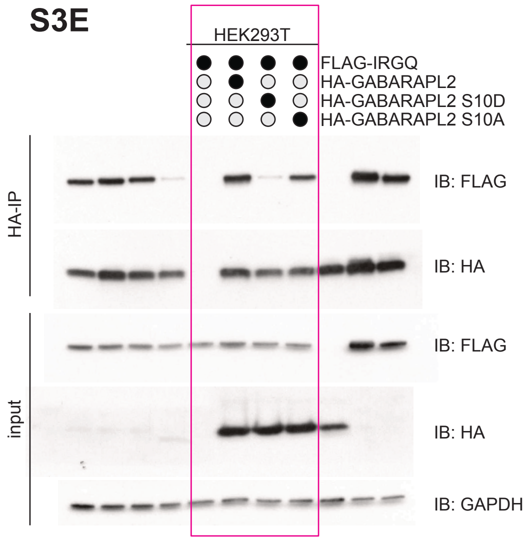

4A

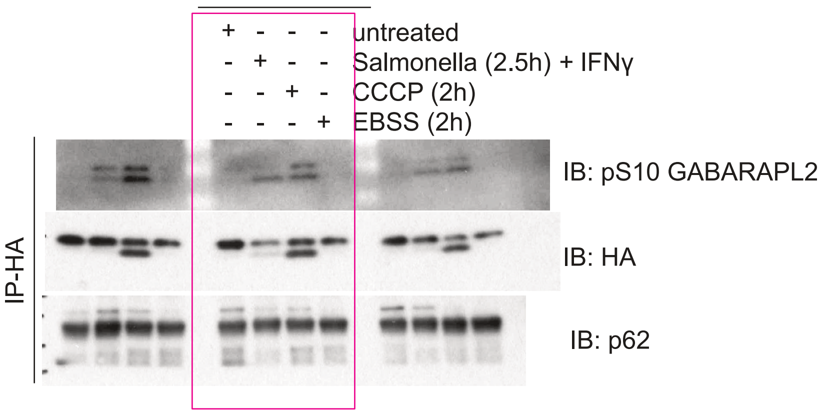

S3G

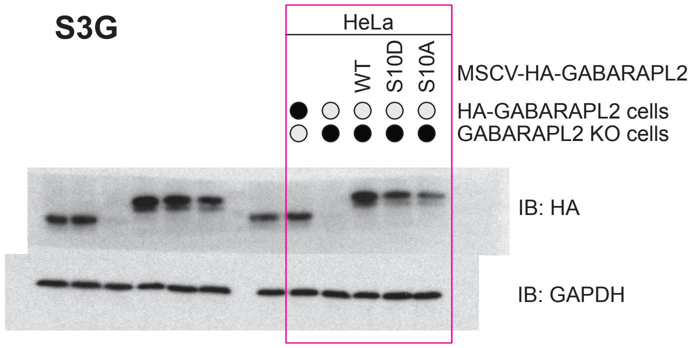

4E

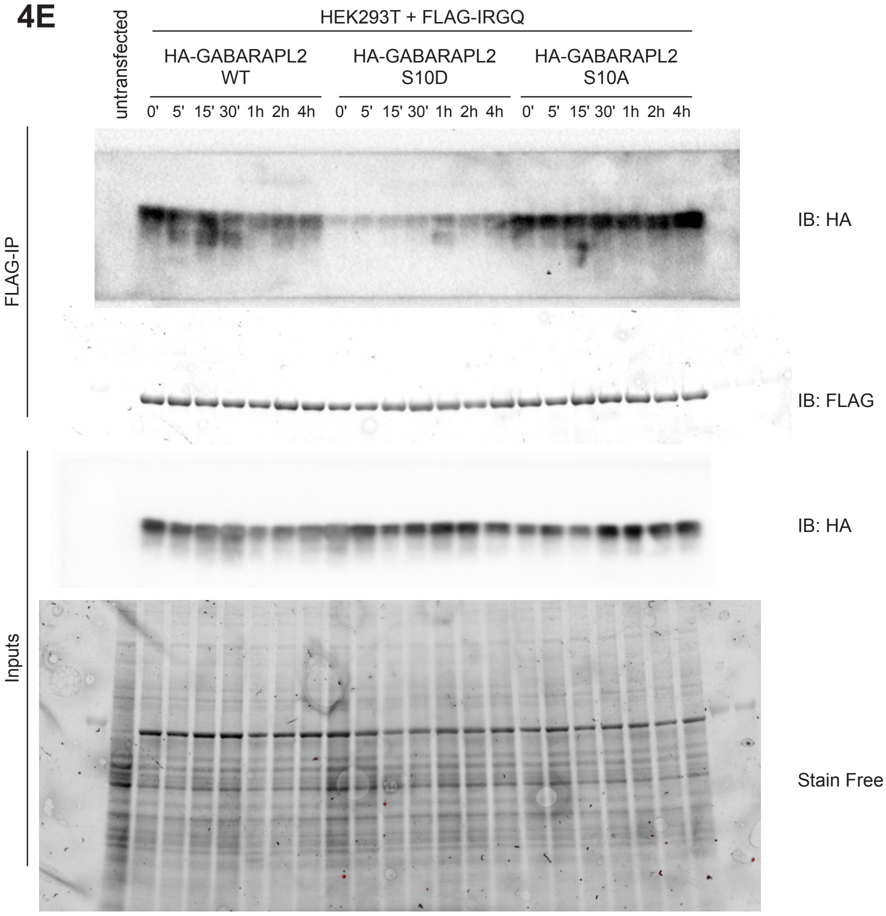

4D

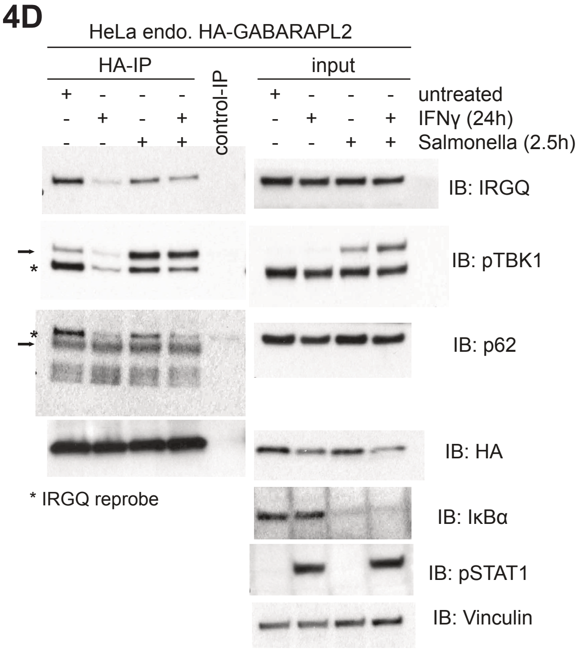

Raw data (4): uncropped blots / membranes

S6A

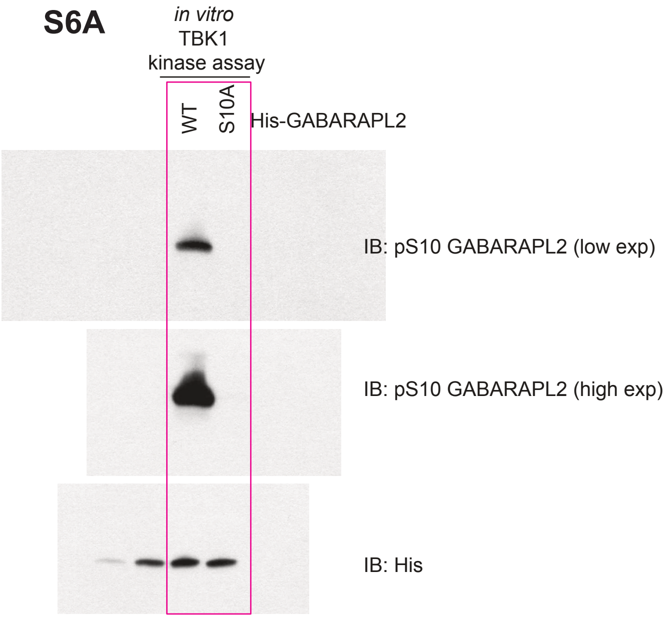

S6D

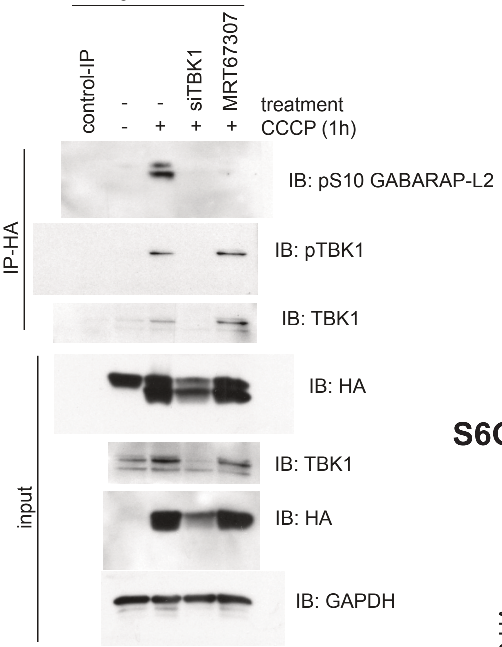

S6E

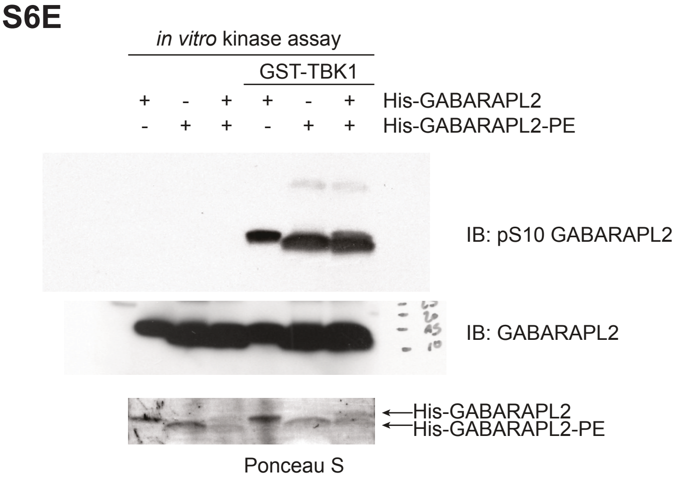

S6G

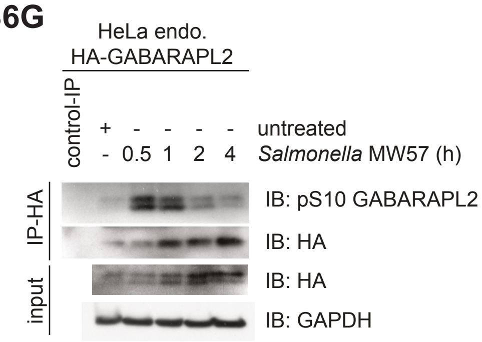

S6F

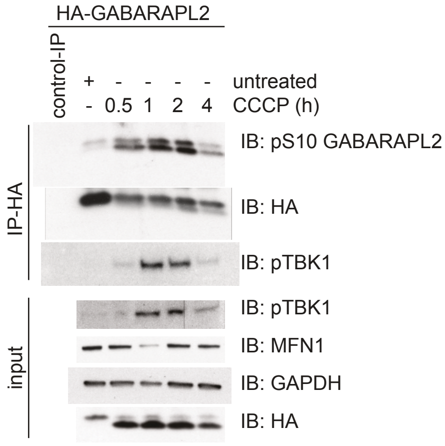

S6K

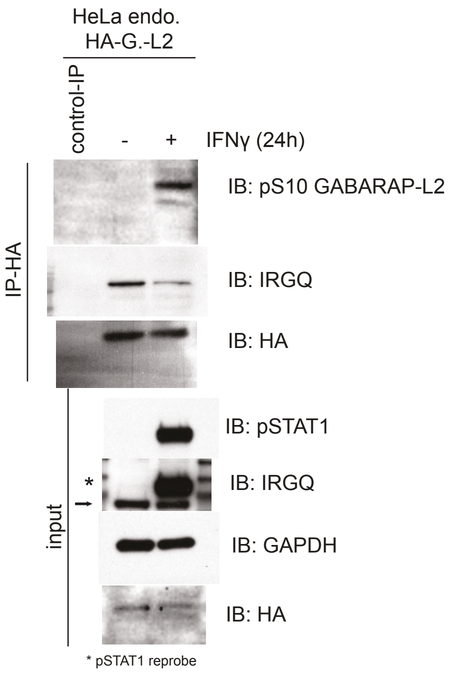

Supplement: Supplementary file 5 — Source Data For Raw Blots [file 41467_2026_73005_MOESM5_ESM.pdf]
